# Detection of Plant DNA in the Bronchoalveolar Lavage of Patients with Ventilator-Associated Pneumonia

**DOI:** 10.1371/journal.pone.0011298

**Published:** 2010-06-24

**Authors:** Sabri Bousbia, Laurent Papazian, Bernard La Scola, Didier Raoult

**Affiliations:** 1 Unité de recherche sur les maladies infectieuses et tropicales émergentes, Faculté de Médecine, CNRS-IRD UMR 6236-198, Marseille, France; 2 Service de Réanimation Médicale, Hôpitaux Sud, Marseille, France; University of Giessen Lung Center, Germany

## Abstract

**Background:**

Hospital-acquired infections such as nosocomial pneumonia are a serious cause of mortality for hospitalized patients, especially for those admitted to intensive care units (ICUs). Despite the number of the studies reported to date, the causative agents of pneumonia are not completely known. Herein, we found by molecular technique that vegetable and tobacco DNA may be detected in the bronchoalveolar lavage from patients with ventilator-associated pneumonia (VAP).

**Methodology/Principal Findings:**

In the present study, we studied bronchoalveolar lavage (BAL) from patients admitted to ICUs with ventilator-associated pneumonia. BAL fluids were assessed with molecular tests, culture and blood culture. We successfully identified plant DNA in six patients out of 106 (6%) with ventilator-associated pneumonia. Inhalation was confirmed in four cases and suspected in the other two cases. Inhalation was significantly frequent in patients with plant DNA (four out of six patients) than those without plant DNA (three out of 100 patients) (*P*<0.001). *Nicotiana tabacum* chloroplast DNA was identified in three patients who were smokers (cases 2, 3 and 6). *Cucurbita pepo*, *Morus bombycis* and *Triticum aestivum* DNA were identified in cases 1, 4 and 5 respectively. Twenty-three different bacterial species, two viruses and five fungal species were identified from among these six patients by using molecular and culture techniques. Several of the pathogenic microorganisms identified are reported to be food-borne or tobacco plant-associated pathogens.

**Conclusions/Significance:**

Our study shows that plants DNA may be identified in the BAL fluid of pneumonia patients, especially when exploring aspiration pneumonia, but the significance of the presence of plant DNA and its role in the pathogenesis of pneumonia is unknown and remains to be investigated. However, the identification of these plants may be a potential marker of aspiration in patients with pneumonia.

## Introduction

Hospital-acquired pneumonia represents a serious cause of mortality for patients admitted to intensive care units (ICUs), especially for those receiving mechanical ventilation [1]. Aspiration pneumonia may occur as a consequence of the passage of foreign material such as gastric or oropharyngeal contents, into the respiratory tracts. It has been reported that aspiration of foreign matter may occur easily among ICU patients due to several factors including the patient's body position and the use of enteric feeding and mechanical ventilation [2]–[6]. This type of pneumonia may represent a serious cause of illness and mortality among ICU patients [7], [8]

However, the causative agents of pneumonia have not been completely identified [9]. Therefore, different strategies have been used to complete an identification of the repertoire of microbes associated with pneumonia. Recently, several studies have been performed using 16S rDNA amplification and sequencing to identify bacteria in the sputum of patients with cystic fibrosis or pneumonia. These studies show that most of the bacteria found had not previously been identified [10]–[13]. Recently, we applied the same technique in patients hospitalized with pneumonia in ICUs and identified *Tropheryma whipplei* as a single agent or in mixed flora of oral origin [14]. Moreover, viral communities have also been reported in patients with cystic fibrosis [15].

Herein, when conducting a large study of pneumonia agents in patients with ventilator-associated pneumonia, we were surprised to identify DNA from food plants and tobacco that had been inhaled by patients undergoing mechanical ventilation in the ICU.

## Results

This study was a part of a very large project implemented in our laboratory in order to perform an exhaustive etiologic diagnosis of pneumonia. Herein, we focused on six patients out of 106 cases with ventilator-associated pneumonia for which we surprisingly identified plant DNA in their BAL fluids by using PCR. The sex ratio was of these patients was 1∶1 (3 men and 3 women). The patients ranged in age from 43 to 74 years and their clinical data are summarized in Table 1. Inhalation was confirmed in a total of 7 patients out of the 106 cases, of which 4 were positive for plant DNA (57%). Inhalation was suspected in the two other patients positive for plant DNA (Table 1). Statistical analysis showed that inhalation was significantly frequent in patients with plant DNA (four out of six patients) than those without plant DNA (three out of 100 patients) (*P*<0.001). Using PCR (16S and 18S rDNA amplification followed by cloning and sequencing), four plant species, twenty-two different bacterial species and two fungal species were identified (Table 2). In two cases (cases 1 and 5), we identified *Cucurbita pepo* (zucchini), which may have been acquired via inhalation of oral flora contaminated by food. Interestingly, in one case (case 1), *C. pepo* DNA was associated with *Achromobacter xylosoxidans*, a water-borne opportunistic pathogen, and *Candida dubliniensis*. While his antibiotherapy was modified after the pneumonia diagnosis (he received Ticarcillin-clavulanic acid, Linezolide, Tobramycin and Voriconazole), no improvement was observed and he died later. In another case (case 5), the DNA was found along with another plant (*Triticum aestivum*, bearded spring wheat, used for making bread), a bacterium close to *Raoultella planticola* (a plant pathogen), three *Lactobacillus* species and *Candida albicans*. This patient was admitted to the ICU for chronic obstructive lung disease and suspected nosocomial pneumonia. His plant inhalation might be occurred during the hospitalization. For another patient (case 4), DNA from *Morus bombycis* (black mulberry) was found. It was associated with uncultured *Neisseria* sp., *Pseudomonas stutzeri* and *Staphylococcus epidermidis*. Finally, in three other cases DNA which matched 100% with the *Nicotiana tabacum* chloroplast genome (among other species identified) was detected. This DNA may have been introduced by tobacco inhalation before the pneumonia episode in question (the three patients were all smokers). Available Clinical data showed that three of these six plant-positive patients had a history for pneumonia at the admission (cases 2, 5 and 6) (Table 1).

**Table 1:** Summary of clinical and sociodemographic features of patients positive for plant DNA.

*Case*	*Age, Sex*	*SAPS II at admission*	*Cause of admission to ICU*	*Smoker*	*ICU Stay (days)*	*Aspiration*	*Evolution during the 10 days following BAL*	*Therapeutic modifications following diagnosis*	*Outcome*
1	47, F	30	Lung contusion/ARDS/bronchopleural fistulae	No	7	Certain	No improvement	Ticarcillin-clavulanic acid/Linezolide/Tobramycin/Voriconazole	Died
2	45, M	35	Multiple trauma/lung contusion/nosocomial pneumonia/ARDS	Yes	21	Possible	Improvement	Cefotaxime/Gentamycin	Survived
3	57, F	50	Coma/aspiration/ARDS	Yes	30	Certain	Improvement	Acyclovir/Methylprednisolone	Survived
4	54, M	25	Esophagectomy/esophageal fistulae	Yes	4	Possible	Improvement	None	Survived
5	74, M	31	Nosocomial pneumonia/aspiration/chronic obstructive lung disease	Yes, but had stopped	180	Certain	Improvement	None	Survived
6	43, F	45	HIV/CAP/coma	Yes	23	Certain	Improvement	None	Survived

ARDS, acute respiratory distress syndrome; CAP, community-acquired pneumonia; HIV, human immunodeficiency virus; SAPS, simplified acute physiology score; ICU, intensive care unit.

**Table 2:** Summary of the molecular results of patients for whom plant DNA was detected in the bronchoalveolar lavage.

*Case*	*Bacteria (Similarity)*	*Parasite (Similarity)*	*Virus*	*Plant species (Similarity)*	*Primer set*	*Plant names*	*GenBank N°*
1	*Achromobacter xylosoxidans* (99%)	*Candida dubliniensis* (100%)	CMV	*Cucurbita pepo* (100%)	18S rDNA	Zucchini	EF595858.1
2	*Acinetobacter* sp. (96%)*Stenotrophomonas maltophilia* (99%)*Acinetobacter johnsonii* (99%)*Aeromonas hydrophila* (99%)	Negative	Negative	*Nicotiana tabacum* (100%)	16S rDNA	Tobacco plant	Z00044.2
3	*Haemophilus parainfluenzae* (99%)*Pseudomonas fluorescens* (99%)*Pseudomonas stutzeri* (99%)*Actinomyces* genomosp. C2 (99%)	Negative	HSV	*Nicotiana tabacum* (100%)	16S rDNA	Tobacco plant	Z00044.2
4	Uncultured *Neisseria* sp. (99%)*Pseudomonas stutzeri* (99%)*Staphylococcus epidermidis* (99%)	Negative	Negative	*Morus bombycis* (100%)	18S rDNA	Black mulberry	AM042006.1
5	*Lactobacillus gasseri* (99%)*Lactobacillus* sp. (92%)*Pseudomonas aeruginosa* (99%)*Lactobacillus crispatus* (95%)*Raoultella planticola* (94%)	*Candida albicans* (100%)	CMV	*Triticum aestivum* (100%)*Cucurbita pepo* (100%)	18S rDNA18S rDNA	Bread wheatZucchini	EF595858.1
6	*Leptotrichia* sp. (99%)*Enterococcus mundtii* (99%)*Diaphorobacter* sp. (100%)*Stenotrophomonas maltophilia* (100%)*Paracoccus thiophilus* (99%)	Negative	Negative	*Nicotiana tabacum* (100%)	16S rDNA	Tobacco plant	Z00044.2

CMV, cytomegalovirus; HSV, herpes simplex virus.

Quantitative PCR for mimivirus, parainfluenza viruses 1 and 3, respiratory syncytial virus, rhinovirus, metapneumovirus, varicella-zoster virus, influenza viruses A and B, enterovirus and coronaviruses were negative. Two viruses were identified by quantitative PCR. Cytomegalovirus was identified in cases 1 and 4 with food-borne plant DNA, whereas herpes simplex virus was identified in case 3 along with *Nicotiana tabacum*.

Bacterial BAL culture was positive for *Pseudomonas aeruginosa* and *Staphylococcus aureus* in case 5 (Table 3). Fungal BAL culture identified five fungal species that may not be involved with the pneumonia but, rather, be contaminants. DNA from both *N. tabacum* and *Aspergillus fumigatus* was identified in case 2. This patient was admitted to the ICU for multiple trauma, lung contusion and acute respiratory distress syndrome complicating nosocomial pneumonia. Bacterial and fungal blood cultures were negative for all six plant DNA-positive patients (Table 3).

**Table 3:** Bronchoalveolar lavage and blood culture results of patients with plant DNA identified by molecular methodology.

*Case*	*Bacterial culture*		*Fungal culture*	
	BAL culture	Blood culture	BAL culture	Blood culture
1	Oral flora	Negative	*Candida dubliniensis*	Negative
2	Negative	Negative	*Aspergillus fumigatus*	Negative
3	Negative	Negative	Negative	Negative
4	*Staphylococcus epidermidis*	Negative	*Candida albicans*	Negative
5	*Pseudomonas aeruginosa* *Staphylococcus aureus*	Negative	*Candida albicans* *Candida krusei*	Negative
6	Negative	Negative	*Candida glabrata*	Negative

## Discussion

Aspiration of foreign oropharyngeal material or gastric content is a serious risk factor for the development of aspiration pneumonia in patients receiving mechanical ventilation in the ICU. Our work shows that plant DNA can be found when investigating aspiration pneumonia episodes using 16S (chloroplast) and 18S rDNA (chromosome) amplification and sequencing. Our results are in agreement with previously reported studies. In fact, in a previous study, Crome *et al.* reported that inhaled vegetable particles may cause pulmonary nodular granulomatosis [16]. Moreover, in one recent study reported by Mukhopadhyay *et al.*, the authors demonstrated histologically that vegetable material was the most common foreign substance identified in 92% of biopsies from patients with aspiration pneumonia [17]. They showed that the vegetable matter is frequently located within airspaces and surrounded by multinucleated giant cells or neutrophils. They also reported that, in some cases, more than one of the various forms of vegetable particles were observed together, similar to one of our patients (case 5), in whom two plants were identified (*T. aestivum* and *C. pepo*) along with a bacterium close to *R. planticola* (a plant pathogen), *P. aeruginosa* and *S. aureus*, which are pathogenic bacteria widely associated with pneumonia. Previously, studies of patients with pulmonary infection and cystic fibrosis or no underlying disease identified chloroplast DNA using 16S rDNA amplification and sequencing [11]. It has also been reported that we inhale billions of particles every day, including plant pollen [18]. Additionally, in one recent study, Sapkota *et al.* studied the bacterial metagenome of several kinds of cigarettes using 16S rRNA-based taxonomic microarray, cloning and sequencing and found in many clones *Nicotiana tabacum* chloroplasts DNA. This confirms that using broad-range primers to amplify bacterial 16S rDNA gene can detect this tobacco chloroplasts. In our study *N. tabacum* chloroplast DNA was identified in three smokers; aspiration was clinically confirmed in two patients (case 3 and 6) and highly suspected in the third (case 2). Several bacteria detected in the BAL fluids from our positive patients are considered to be part of the oral flora, which indicates that the plant DNA identified herein was inhaled from the oropharyngeal cavity. Most of these bacteria were identified by molecular methodology and were absent using standard culture, especially in patients 1, 2, 3 and 6, which suggests that the molecular identification is clinically useful than culture techniques.

Altogether, this work describes the first association between plant DNA and pneumonia by molecular methodology. This association may be a consequence of the route of infection and the very long persistence of inhaled plant DNA. This is apparently the case for the three smokers, who were hospitalized for more than three weeks without smoking. The presence of DNA from two plants and a probable plant pathogen in case 5 suggests that the acquisition of pneumonia in that case may have been due to inhalation of plant-based food from the oral cavity after a meal. Finally, the identification of food plant DNA in pneumonia patients may be suggestive of inhalation-based pneumonia.

In summary, our study reports plant DNA in aspiration pneumonia among mechanically ventilated patients. Plants were identified in the BAL fluids along with oral bacterial flora and plant- associated bacteria. The long persistence of plant DNA in the lungs of patients hospitalized in the ICU is puzzling despite the fact that there is no evidence of a pathogenic role. Even though research on these plants using molecular techniques may help to confirm the diagnosis of aspiration pneumonia, we cannot draw conclusions as to the potential role of the plants in the pneumonia.

## Methods

### Patients and clinical samples

Bronchoalveolar lavage (BAL) fluids were collected from ICU on patients receiving mechanical ventilation. A total of 106 BAL and 106 blood samples were studied from 106 episodes of ventilator-associated pneumonia in ICU patients. The diagnoses of ventilator-associated pneumonia and aspiration pneumonia were defined as described elsewhere [19], [20]. Written consent from patient family members was obtained. BAL and blood sampling were performed as described elsewhere [21], and the specimens were sent to the microbiology laboratory of Timone Hospital in Marseille, France, where samples were tested. Fungal and blood cultures were performed in the parasitology laboratory of Timone Hospital in Marseille, France. BAL samples were tested by molecular biology, and standard culture approaches were followed to identify the bacteria, fungi and viruses present in the samples. Blood specimens were tested only by blood culture. The project was approved by the Local Ethics Committee of the Université de la Méditerranée (Marseille, France), with the permit number: 07-026.

### Nucleic acid extraction

Bacterial and fungal DNA was extracted from BAL samples using a MagNA Pure LC workstation (Roche Diagnostics, Meylan, France) with the MagNa Pure LC DNA Isolation Kit II (Roche Diagnostics). A mixture of 200 µL of lysis buffer and 50 µL of proteinase K weas mixed with BAL pellets and incubated overnight at 56°C. The pellets were then disrupted for 1 min using glass beads in a MagNa Lyser (Roche Diagnostics) and processed on the automatic workstation following the supplier's recommendations. When the BAL was cell-poor, 500 µL of BAL sample was centrifuged for 15 minutes at maximal speed. Then, 400 µL of the supernatant was discarded and the pellet was then resuspended in the remaining 100 µL µL to concentrate the bacterial cells. The extraction procedure was then continued as described above. Viral nucleic acids were extracted from 200 µL of BAL fluids using an MDX workstation and QIAamp Virus BioRobot MDx Kit, as recommended by the supplier.

### PCR amplification, cloning and sequencing

DNA was tested by PCR for universal bacteria using broad-range primers to amplify the 16S rDNA gene and tested for universal fungi using broad-range primers to amplify 18S rDNA (16S rDNA primer set: 536F 5′-CAGCAGCCGCGGTAATAC-3′ and rp2 5′-ACGGCTACCTTGTTACGACTT-3′; 18S rDNA primer set: UC18SF 5′-TCCGTAGGTGAACCTGCGG-3′ and UC 18SR 5′-GCTGCGTTCTTCATCGATGC-3′) (Eurogentec, Seraing, Belgium). PCR products were cloned and sequenced.

PCR was performed using an ABI Thermocycler (Applied Biosystems, Courtaboeuf, France). Each amplification was carried out in a final volume 50 µL containing 5 µL of extracted DNA, 1× PCR buffer, 2 µL of 25mM MgCl2, 200 µM of each dNTP, 0.2 mM of each primer and 1 unit of HotStar Taq DNA Polymerase (QIAGEN, Courtaboeuf, France). Amplification was started with an initial incubation at 95°C for 15 min, followed by 35 cycles of heating at 95°C for 1 min, annealing for 30 s (62°C for the 16S rDNA primer set, and 60°C for the 16S rDNA primer set) and extension at 72°C for 90 s. Amplification was concluded with an incubation step at 72°C for 10 min. The Nucleo-Fast 96 PCR Kit (MACHEREY-NAGEL, Hoerdt, France) was used to purify the PCR reactions as described by the manufacturer. Purified PCR products from the previous steep were cloned into a PCR II TA cloning vector (Invitrogen, Cergy Pontoise, France), following the manufacturer's recommendations. White colonies were screened from LB-agar plates and the cloned inserts were amplified with the M13 primer set (M13F: 5′-GTAAAACGACGGCCAG-3′, M13R: 5′-CAGGAAACAGCTATGAC-3′) and sequenced in 20 µL final volume containing 1× sequencing buffer, 3.2 pmol of forward (536F) or reverse (rp2) primer, 3 µL of Big Dye Terminator V1.1 mix (Applied Biosystems) and 8 µL of de-ionized water. Sequencing reactions were purified using Sephadex Gel Filtration (Sigma-Aldrich Chimie, Saint Quentin Fallavier, France) and the purified products were sequenced on an ABI PRISM 3130xl genetic sequencer (Applied Biosystems). Subsequently, the obtained sequences were assembled and analyzed by Chromaspro software and then compared with those available in the GenBank database (www.ncbi.nlm.nih.gov) by BLAST search in order to identity of the corresponding species.

### Quantitative PCR for virus detection

Mimivirus, cytomegalovirus, herpes simplex virus, parainfluenza viruses 1and 3, respiratory syncytial virus, rhinovirus, metapneumovirus, varicella-zoster virus, influenza viruses A and B, enterovirus and coronaviruses OC-43, 229-E and NL-63 were tested for by using quantitative PCR (qPCR). Quantitative PCR was performed using a LightCycler® instrument (Roche Diagnostics, Meylan, France) with the QuantiTect Probe PCR Kit. Primers and probes used to identify viruses are listed in Table 4. Each qPCR reaction was carried out in a final volume of 20 µL containing 10 µL of QuantiTect master mix, 2 µL of probe 0.1 mM of each primer and 4 µL of DNA. Quantitative PCR was was initiated with an enzyme-activation incubation at 95°C for 15 min to activate DNA polymerase, followed by 40 cycles of denaturation at 95°C for 10 s and an annealing-extension step at 60°C for 1 min. For RNA viruses, RNA was first reverse transcribed. RNA reverse transcription was performed in a final volume of 25 µL containing 1.5 µg of total nucleic acids, 1× RT Buffer, 5.5 µL of MgCl_2_ Solution, 5 µL of dNTP mixture and 0.5 µL of RNase inhibitor. The reaction mix was incubated for 2 min at 70°C in order to disrupt secondary RNA structure and then chilled on ice. Subsequently, 400 µM of random hexamer primers and 40 units of MultiScribe™ Reverse Transcriptase (Applied Biosystems, Courtaboeuf, France) were added. The reaction mixture was incubated at 25°C for 10 min and then at 40°C for 90 min in an ABI Thermocycler (Applied Biosystems). Quantitative PCR was continued as described above.

**Table 4:** Primers and probes used in viral quantitative PCR.

*Virus*	*Gene*	*Forward primer*	*Reverse primer*	*Probe*
Mimivirus	Capside	5′-GATAAACATTATGGTGACTG-3′	5′-AGGAACATACAGAGTATATG-3′	5′-ATCATGAAAAGGGTCTTGCTA-3′
RSV A	Gene n	5′-AGATCAACTTCTGTCATCCAGCAA-3′	5′-GCACATCATATTTAGGAGTATCAAT-3′	5′-CTTTGCCATACTCAATGAACAAAC-3′
RSV B	Gene n	5′-AAG TGCAAATGATAAATTCACAGGA-3′	5′-TAGTATCCAGCATCTTTAAGTZTCTTTATAG-3′	5′-CACCATCCAACGGAGCAGAGGAGAT-3′
influenza A	Gene m	5′-GGACTGCAGCGTAGACGCTT-3′	5′-CATYCTGTTGTATATGAGGCCCAT-3′	5′-CTCAGTTATTCTGCTGGTGCACTTGCCA-3′
influenza B	Gene h	5′-AATACGGTGGATTAAATAGCAA-3′	5′-CCAGCAATAGCTCCGAAGAAA-3′	5′-CACCCATATTGGGCAATTTCCTATGGC-3′
Parainfluenza 1	Hg/Ne	5′-CATTATCAATTGGTGATGC-3′	5′-CTTAAATTCAGATATGTATCCTG-3′	5′-CTTAATCACTCAAGGATGTGCAGATATA-3′
Parainfluenza 3	Hg/Ne	5′-CTCGAGGTTGTCAGGATATAG-3′	5′-CTTGGGAGTTGAACACAGTT-3′	5′-AATAACTGTAAACTCAGACTTGGTACCTGACTT-3′
Rhinovirus	5′ NCR	5′-GCACTTCTGTTTCCCC-3′	5′-GGCAGCCACGCAGGCT-3′	5′-AGCCTCATCTGCCAGGTCTA-3′
Metapneumovirus	Gene N	5′-AACCGTGTACTAAGTGATGCACTC-3′	5′-CATTGTTTGACCGGCCCCATAA-3′	5′-CTTTGCCATACTCAATGAACAAAC-3′
Enterovirus	5′NC	5′-CCCTGAATGCGGCTAATCC-3′	5′-ATTGTCACCATAAGCAGCCA-3′	5′-CADGGACACCCAAAGTAGTCGGTTCC-3′
Coronavirus OC-43	Pol	5′-CGCCGCCTTATTAAAGATGTTG-3′	5′-GGCATAGCACGATCACACTTAGG-3′	5′-AATCCTGTACTTATGGGTTGGGATT-3′
Coronavirus 229-E	Pol	5′-TGGAGCGAGGATCGTGTTC-3′	5′-TAGGCTGTGACAGCTTTTGCA-3′	5′-TGTTCTCACGCTGCTGTTGATTCGCT-3′
Coronavirus NL-63	Replicase	5′-TGTTGTAGTAGGTGGTTGTGTAACATCT-3′	5′-AATTTTTGTGCACCAGTATCAAGTTT-3′	5′-ATGTTTCACCAATTGTTAGTGAGAAAATTTCTGTTATGGA-3′
HSV	Pol	5′-CATCACCGACCCGGAGAGGGAC-3′	5′-GGGCCAGGCGCTTGTTGGTGTA-3′	5′-CCGCCGAACTGAGCAGACACCCGCGC-3′
VZV	Pol	5′-GGTTAAACGTTTGAATCCATCC-3′	5′-CAGCAGACTTTCTCGAACGT-3′	5′-ATGCCACCTTTACAGTTGGAGGAA-3′
CMV	pp65	5′-GCAGCCACGGGATCGTACT-3′	5′-GGCTTTTACCTCACACGAGCATT-3′	5′-CGCGAGACCGTGGAACTGCG-3′

RSV, respiratory syncytial virus; VZV, varicella-zoster virus; CMV, cytomegalovirus; HSV, herpes simplex virus.

### Culture, blood culture and phenotypic identification

Bacteriological BAL culture, blood culture and phenotypic identification were performed as previously described [13], [22]. For bacterial BAL culture, we used three different media: chocolate Poly ViteX agar, MacConkey agar and blood agar. All growth media were purchased from bioMérieux (bioMérieux, Marcy l'Etoile, France). Media were incubated at 37°C for 48 h, and then the colonies growing on the various media were identified using Gram staining, an API system (bioMérieux, Marcy l'Etoile, France), a VITEK 2 Auto system (bioMérieux, Marcy l'Etoile, France) and standard procedure for antibiotic susceptibility testing. For bacterial blood culture, blood samples were taken in Bactec Plus aerobic and Lytic 10 anaerobic blood culture bottles and processed using the automate Bactec 9240 system according to the manufacturer's recommendations. When a blood culture sample was detected as positive, the blood culture broth was deposited on a glass slide, Gram stained and and subcultured onto chocolate Poly ViteX and Columbia sheep blood agar (bioMérieux, Marcy l'Etoile France) under aerobic and anaerobic conditions. A 10^4^ colony forming unit (CFU) cut-off point was used to identify positive cultures. Identification of fungi present in BAL or blood samples was performed using conventional culture procedures as previously described [23], [24].

### Statistical analysis

Statistical analysis was performed using Chi2 test. P value was considered significative is below or equally to 0.05.
