# Expression of Concern: Detection of Plant DNA in the Bronchoalveolar Lavage of Patients with Ventilator-Associated Pneumonia

**DOI:** 10.1371/journal.pone.0348163

**Published:** 2026-04-28

**Authors:** The PLOS One Editors

Following the publication of the article PLOS investigated concerns pertaining to the reported ethical approval and the article’s adherence to *PLOS One*’s research ethics policies.

Specifically, the research ethics concerns included that the study involved human participants but did not receive ethics approval from a Comité de Protection des Personnes, and that the ethics approval number #07–026 was also reported in multiple other publications [2–4], [5–7], and [8,9, retracted in 10], despite differences in the requirements for consent reported in these articles.

The study describes the use of bronchoalveolar lavage and blood samples collected from intensive care unit patients with ventilator-associated pneumonia, but does not clarify when these samples were collected. The article describes that consent was obtained from next-of-kin, and that the project was approved by the Local Ethics Committee of the Université de la Méditerranée (Marseille, France), with the permit number: 07–026.

A representative of the Aix-Marseille Université Ethics Committee stated that the institutional investigation into the ethics concerns concluded this article meets ethical standards. They commented that the study described in [1] is a retrospective study of samples taken in intensive care units as part of routine patient care, and that the study did not require ethics approval from a Comité de Protection des Personnes according to French law. Furthermore, the representative stated that these articles resulted from the same retrospective study and therefore the same ethics approval document applied to all articles. They provided the ethics approval document #07–026 for editorial review.

Ethics approval document #07–026 was issued by the Comité d'Éthique de l'Institut Fédératif de Recherché de la Faculté de Médecine de Marseille (IFR48, Marseille, France) on February 19, 2007, for a study titled: “*Recherche de nouveaux agents de pneumopathies nosocomiales par une approche métagénomique différentielle.*” It allows for the use of blood, urine, and respiratory samples collected for diagnostic purposes, but the document does not clarify where the samples were collected or for how long the approval remained valid.

PLOS reviewed the explanation and documentation provided by the institution and concluded that the documents did not fully resolve the concerns. Specifically,

- If all articles were part of the same larger study, concerns remain about inconsistencies between the reporting of the study design in the articles’ Methods/Materials and Methods sections (no reporting in [1] and [2–4] and reported as a prospective study in [5–7] and [8,9 retracted in 10]), and the institute’s reporting of the study design (retrospective).
- If all articles were part of the same larger study, concerns remain about inconsistencies between the reporting of consent obtained for the study in each article (written consent obtained from next-of-kin reported in [1], [2–4], and [5–7], need for written consent waived by the ethics committee [8,9, retracted in 10]), a statement that there are no written consents for this study previously provided by the institute [10], and the information in the ethics document #07–026 stating that patient consent was obtained.
- PLOS identified potential competing interests between multiple members of the approving ethics committee and one or more of the article’s authors.

PLOS has not been able to secure input from an independent official confirming whether or not this study should have been subjected to CPP approval as per French legislation.

The *PLOS One* Editors issue this Expression of Concern to notify readers of the concerns pertaining to the article’s compliance with the journal’s research ethics policies.
